# Travel Volume to the United States from Countries and U.S. Territories with Local Zika Virus Transmission

**DOI:** 10.1371/currents.outbreaks.ac6d0f8c9c35e88825c1a1147697531c

**Published:** 2016-05-31

**Authors:** Bradley Nelson, Stephanie Morrison, Heather Joseph, Abbey Wojno, R. Ryan Lash, Yoni Haber, Andre Berro, Martin Cetron, Ardath Grills

**Affiliations:** Eagle Medical Services, LLC for Division of Global Migration and Quarantine, Centers for Disease Control and Prevention, Atlanta, Georgia, USA; Eagle Medical Services, LLC for Division of Global Migration and Quarantine, Centers for Disease Control and Prevention, Atlanta, Georgia, USA; Division of Global Migration and Quarantine, Centers for Disease Control and Prevention, Atlanta, Georgia, USA; Karna, LLC for Division of Global Migration and Quarantine, Centers for Disease Control and Prevention, Atlanta, Georgia, USA; Division of Global Migration and Quarantine, Centers for Disease Control and Prevention, Atlanta, Georgia, USA; Eagle Medical Services, LLC for Division of Global Migration and Quarantine, Centers for Disease Control and Prevention, Atlanta, Georgia, USA; Division of Global Migration and Quarantine, Centers for Disease Control and Prevention, Atlanta, Georgia, USA; Division of Global Migration and Quarantine, Centers for Disease Control and Prevention, Atlanta, USA; Departments of Medicine and Epidemiology, Emory University School of Medicine and Rollins School of Public Health, Atlanta, Georgia, USA; Division of Global Migration and Quarantine, Centers for Disease Control and Prevention, Atlanta, Georgia, USA

## Abstract

Introduction: Air, land, and sea transportation can facilitate rapid spread of infectious diseases. In May 2015 the Pan American Health Organization (PAHO) issued an alert regarding the first confirmed Zika virus infection in Brazil. As of March 8, 2016, the U.S. Centers for Disease Control and Prevention (CDC) had issued travel notices for 33 countries and 3 U.S. territories with local Zika virus transmission.

Methods: Using data from five separate datasets from 2014 and 2015, we estimated the annual number of passenger journeys by air and land border crossings to the United States from the 33 countries and 3 U.S. territories listed in the CDC’s Zika travel notices as of March 8, 2016. We also estimated the annual number of passenger journeys originating in and returning to the United States (primarily on cruises) with visits to seaports in areas with local Zika virus transmission. Because of the adverse pregnancy and birth outcomes that have been associated with Zika virus disease, the number of passenger journeys completed by women of childbearing age and pregnant women was also estimated.

Results: An estimated 216.3 million passenger journeys by air, land, and sea are made annually to the United States from areas with local Zika virus transmission (as of March 8). The destination states with the largest numbers of arrivals were Texas (by land) and Florida (by air and sea). An estimated 51.7 million passenger journeys were made by women of childbearing age and an estimated 2.3 million were made by pregnant women.

Conclusion: Travel volume analyses provide important information that can be used to effectively target public health interventions as well as direct public health resources and efforts at local, regional, and country-specific levels.

## INTRODUCTION

Zika virus is a flavivirus transmitted primarily by Aedes species mosquitoes including *Aedes aegypti* and *Aedes albopictus* 1. In addition, Zika virus can pass from a pregnant woman to her fetus and be sexually transmitted by a man to his partners^1^. Though not confirmed, there have also been reports of Zika virus transmission from blood transfusions^1^. In May 2015, the Pan American Health Organization issued an alert regarding the first confirmed Zika virus infections in Brazil^2^. As of March 8, 2016, the U.S. Centers for Disease Control and Prevention (CDC) had issued 36 travel notices for areas with local Zika transmission^3^. The notices primarily advise that pregnant women postpone travel to affected countries and U.S. territories.

As of March 10, 2016, a total of 193 cases of Zika virus disease had been reported in people in the United States who had recently been to an area with local Zika virus transmission^4^. In February 2016, CDC published information on nine pregnant travelers with confirmed Zika virus disease; 10 additional reports of Zika virus disease in pregnant women were under investigation. Confirmed cases were reported among women who had traveled to areas with local Zika virus transmission^5^.

Air, land, and sea transportation can facilitate rapid spread of infectious diseases^6^^,^ 7 ^,^ 8. In the case of Zika, a travel-associated case (imported case) could potentially introduce the virus into the local mosquito population resulting in autochthonous transmission. The risk of sexual transmission also extends beyond areas with active vector-borne transmission. Understanding travel volume and demographics among arrivals into the United States from areas with local Zika virus transmission can be used to plan health education and risk communication interventions as well as other response efforts in potentially impacted communities and at ports of entry within the United States. We report on the top origins and destinations of passenger journeys between the United States and areas with local Zika virus transmission. We present the number of arrivals to the United States via air, land, and sea from areas with local Zika virus transmission. We then provide estimates of the number of women, women of childbearing age (15-44 years), and pregnant women completing passenger journeys to the United States from areas with local Zika virus transmission. Given growing evidence of a link between Zika virus infection and adverse pregnancy and birth outcomes, these estimates may help identify at-risk populations.

## DATA AND METHODS

For this analysis, areas of interest include the 33 countries and 3 U.S. territories with Zika virus travel notices issued by CDC as of March 3, 2016: American Samoa, Aruba, Barbados, Bolivia, Bonaire, Brazil, Cape Verde, Colombia, Costa Rica, Curacao, Dominican Republic, Ecuador, El Salvador, French Guiana, Guadeloupe, Guatemala, Guyana, Haiti, Honduras, Jamaica, Marshall Islands, Martinique, Mexico, Nicaragua, Panama, Paraguay, Puerto Rico, Saint Vincent and Grenadines, Saint Martin, Samoa, Sint Maarten, Suriname, Tonga, Trinidad and Tobago, U.S. Virgin Islands and Venezuela.

This analysis draws from five separate datasets including Data In, Intelligence Out, Market Intelligence, Fares and Market Sizes, Global LLC (Diio Mi FMg, https://www.diio.net); the U.S. Department of Transportation Origin and Destination Survey data as analyzed by Diio (O&D); the Bureau of Transportation Statistics Border Crossing/Entry Data (http://1.usa.gov/1nJpCkg); U.S. Customs and Border Protection (CBP) Cruise and Charter Data; and CBP Demographics by Mode of Transportation Data.

Using these datasets, we estimated the annual number of passenger journeys by air and land border crossings to the United States and U.S. territories from the 33 countries and 3 U.S. territories listed in the CDC’s Zika travel notices as of March 3, 2016. We also estimated the annual number of passenger journeys from and returning to the United States (primarily on cruises) with visits to seaports in areas with local Zika virus transmission. Because of the adverse fetal and reproductive outcomes that have been linked with Zika virus disease, the numbers of passenger journeys completed by women of childbearing age and pregnant women were also estimated.

Travel to the United States includes travel to all 50 states, the District of Columbia, and all U.S. territories. Gender and age distributions of arrivals by geographic region were calculated using fiscal year 2015 data from CBP. The CBP dataset included information on age, gender, origin, nationality, and mode of transportation for arrivals into the U.S. from countries with local Zika virus transmission.

All CBP records are aggregated passenger counts; therefore, one person might be counted and reported multiple times. For example, arrivals entering the United States from the Mexico border might do so on a daily basis for work or school.

To estimate the number of passenger journeys taken by pregnant women, we first separated all of the data by mode of transportation and then calculated the percentages of women and women of childbearing age (15-44 years old) by region (i.e. South America, North America, Caribbean, Central America, Africa, Oceana) for each mode of transportation. For all modes of transportation, we estimated that 4.5% of female arrivals between the ages of 15-44 are pregnant at any time. The rate of 4.5% was determined by using an estimate of 5% of women pregnant at any time and adjusting for restrictions on travel after 36 weeks gestation^9^^,^ 10 ^,^ 11.

**Air Travel Data**

Air travel data from countries with local Zika virus transmission were obtained from Diio Mi FMg for January 2015 through December 2015. For origins in U.S. territories with documented Zika virus transmission (Puerto Rico, U.S. Virgin Islands and American Samoa), we used the O&D dataset for October 2014 through September 2015. The timeframes between the two datasets differed because of different release dates. Reported counts for both datasets exclude crew members. If an origin or destination city had multiple airports, data from all airports for the city were pooled. For example, data from John F. Kennedy International Airport and La Guardia International Airport were combined into New York, New York data. Final destination airport city indicates the end of the aviation trip, and does not necessarily indicate where travelers end their overall journey. We calculated the percentage of arrivals by women and arrivals by women of childbearing age by region using CBP data because not all regions have the same demographics of air arrivals.

**Land Travel Data**

Land travel data from Mexico into the United States was obtained from the U.S. Department of Transportation, Bureau of Transportation Statistics for January 2014 through December 2014. Arrival counts included travel by personal vehicle, foot, bus, and train. CBP age and gender distributions were applied to arrival counts to estimate the number of arrivals by women and women of childbearing age.

**Sea Travel Data**

Seaport travel information, which includes cruise and charter data, was obtained from CBP for October 2014 through September 2015. This information differs from air and land travel data because seaport passenger journeys originate at a U.S. seaport, travel to a country or U.S. territory with local Zika virus transmission, and end in the same origin U.S. seaport.

## RESULTS

**Air Travel**

For countries with local Zika transmission, Diio FMg data used were from January through December 2015. For U.S. territories with local Zika transmission, DOT O&D data was used from October 2014 through September 2015. The data were merged to create an aggregated 12-month dataset, including information for both countries and U.S. territories. For the 12-month time period a total of 34,334,355 passenger journeys by air to the United States from 33 countries and 3 U.S. territories with local Zika virus transmission were reported (Table 1).

**Figure:**
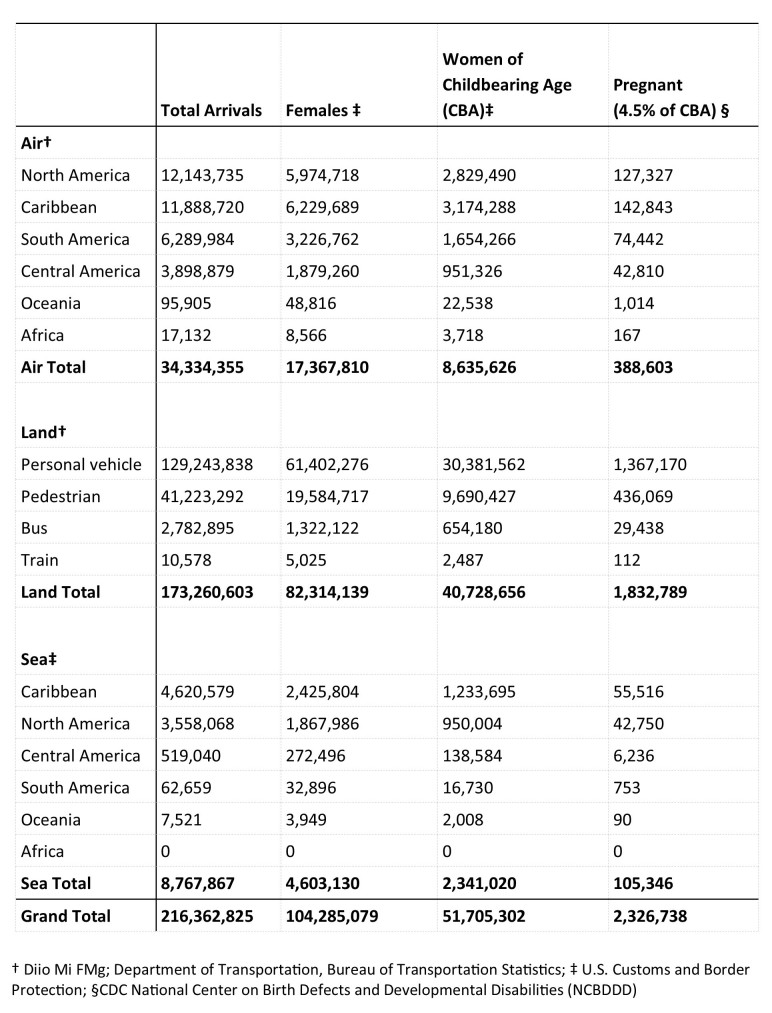
**Table 1.** Passenger Journeys by Air, Land, and Sea to U.S. and U.S. Territories from Countries and U.S. Territories with Local Zika Transmission, 12 Month Estimate

Compared with 2014 data (31,992,504 air passenger journeys), the United States experienced a 2.3 million passenger journey increase. Mexico was the highest volume origin country, accounting for 12,143,735 (35%) of total air arrivals. The top five origin countries and U.S. territories were Mexico, Puerto Rico, Dominican Republic, Brazil and Jamaica, which together accounted for 69% of the total air arrivals (Table 2).

**Figure:**
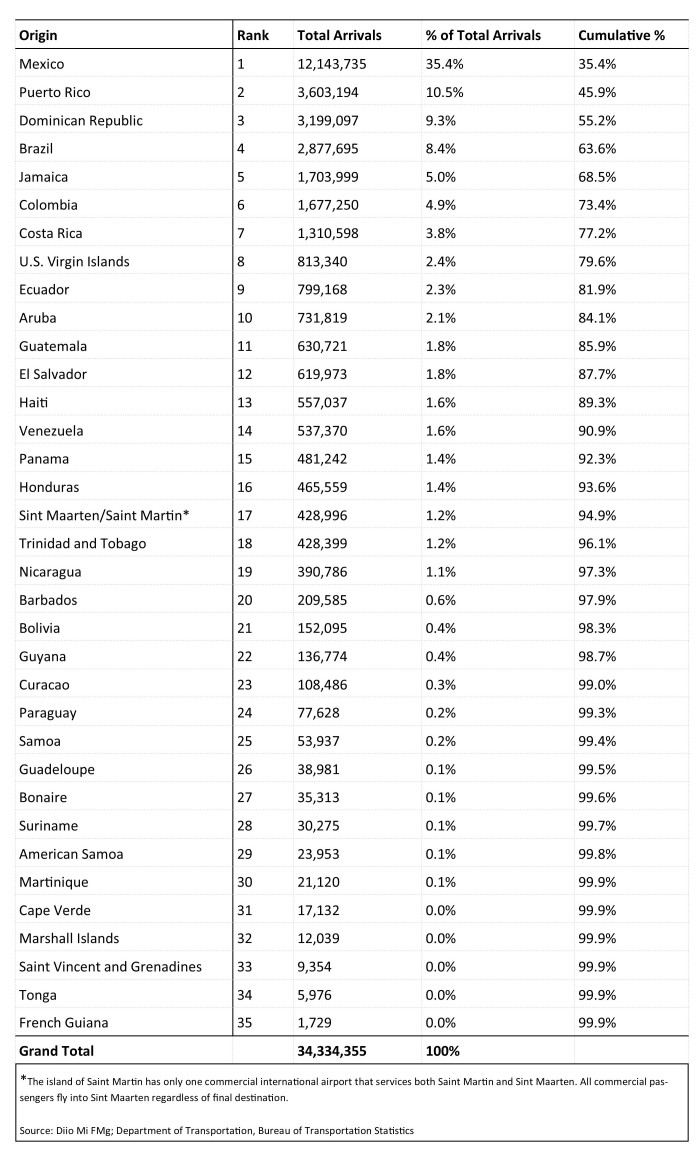
**Table 2.** Aviation Travel to the U.S. from Origins in the 33 Countries and 3 U.S. Territories with Local Zika Transmission, 12 Month Estimate

Three of the top five origin cities were in Mexico (Cancún, Mexico City, Guadalajara) and the top 20 origin cities accounted for 72% of all air arrivals (Table 3).

**Figure:**
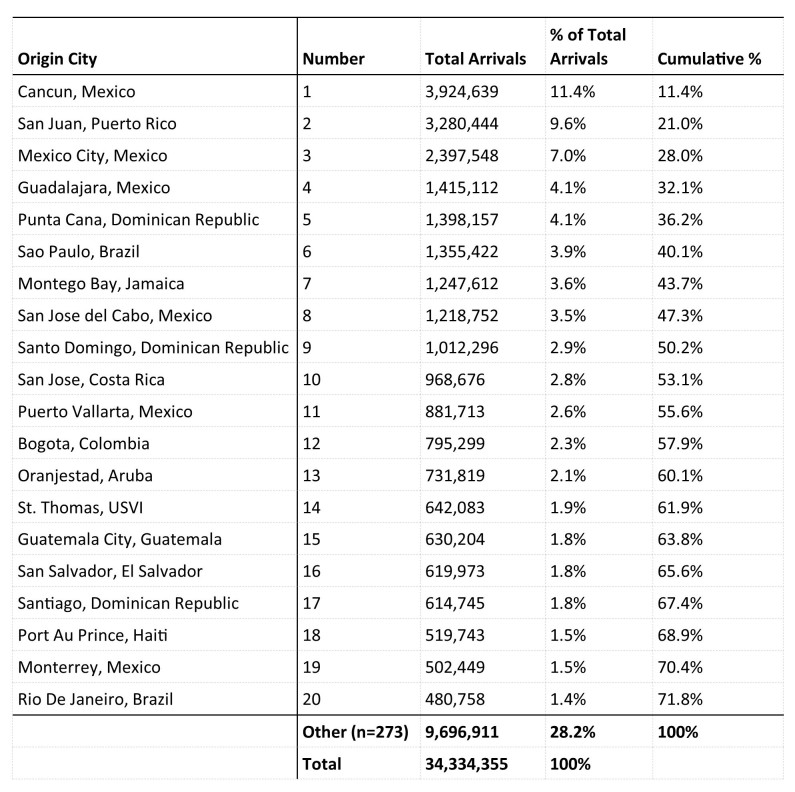
**Table 3.** Top 20 Origin Cities, Aviation Travel to the U.S. from 33 Countries and 3 U.S. Territories with Local Zika Transmission, 12 Month Estimate

Within the United States, New York City, Miami, Los Angeles, Orlando, Fort Lauderdale, Chicago, and Newark were the final destination airport cities, accounting for half of all air arrivals (Table 4).

**Figure:**
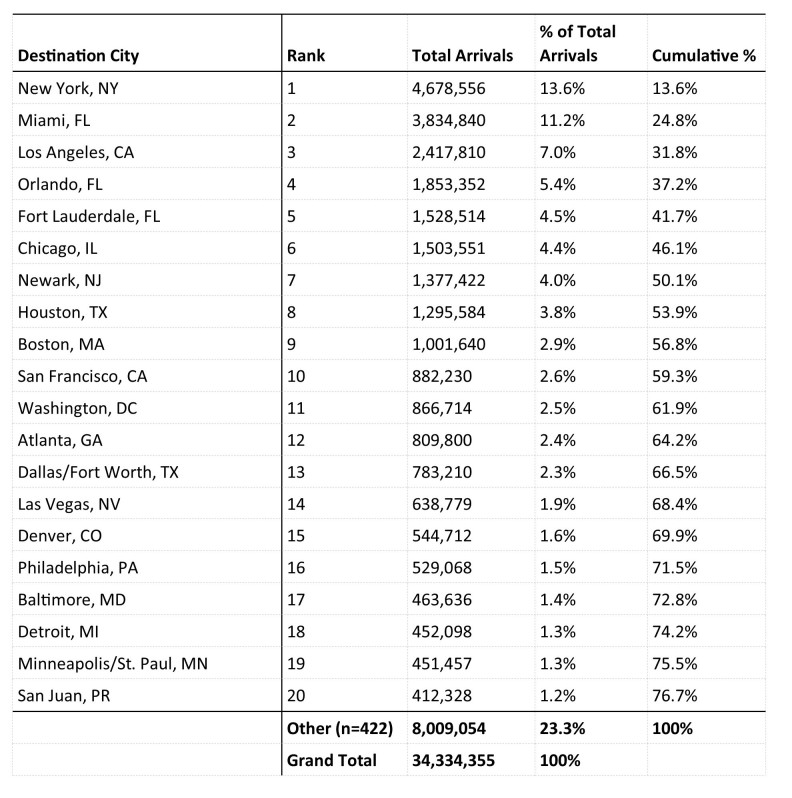
**Table 4.** Top 20 Destination Cities, Aviation Travel to the U.S. from 33 Countries and 3 U.S. Territories with Local Zika Transmission, 12 Month Estimate

New York City was the final destination city that received the largest number of air arrivals (4,678,556), and Florida was the state that received the largest number of air arrivals (7,684,700) (Table 5 and Figure 1).

**Figure:**
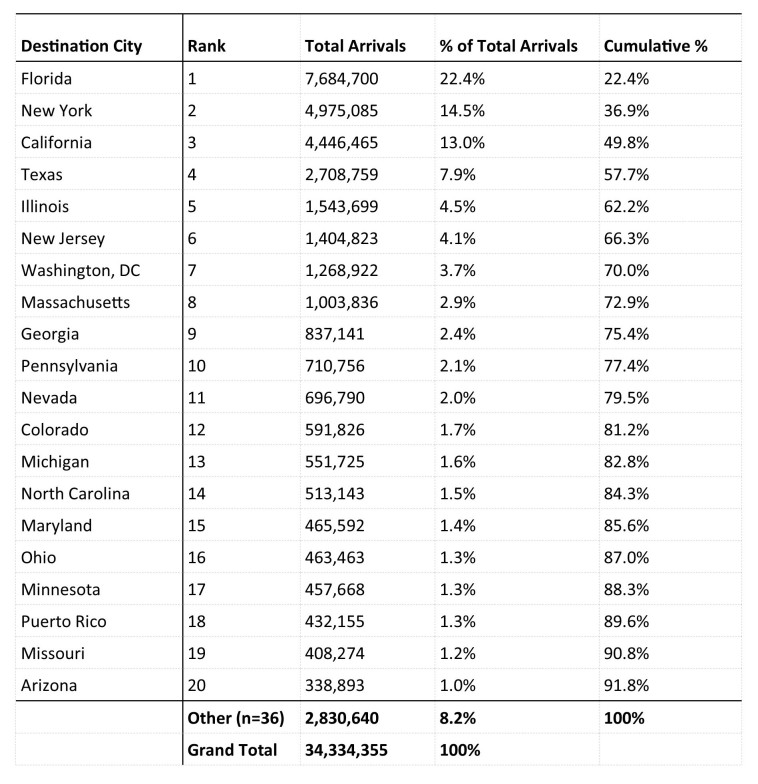
**Table 5.** Top 20 Destination States and U.S. Territories, Aviation Travel to the U.S. from 33 Countries and 3 U.S. Territories with Local Zika Transmission, 12 Month Estimate

**Top 20 Aviation Origin and Destination Cities, 12 Month Estimate:**
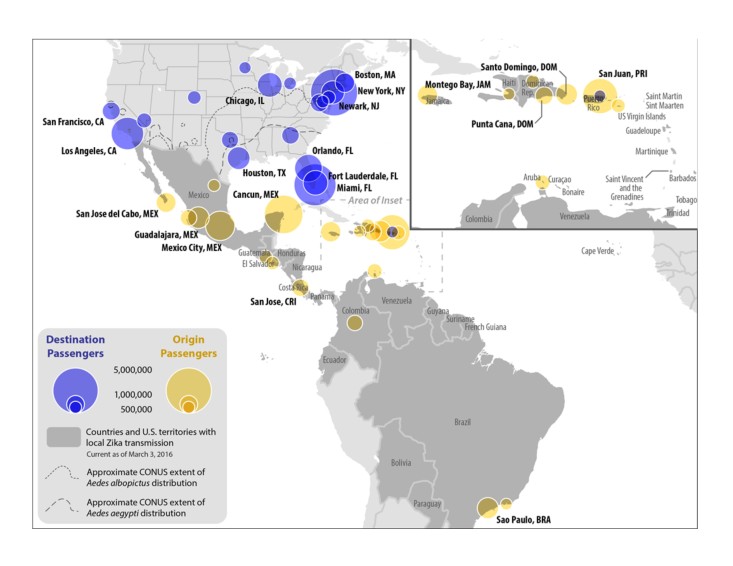

Analysis of CBP demographics data showed between 48-52% of air arrivals into the United States from countries and U.S. territories with local Zika virus transmission were women and 21-27% of air arrivals were women of childbearing age (Table 6).

**Figure:**
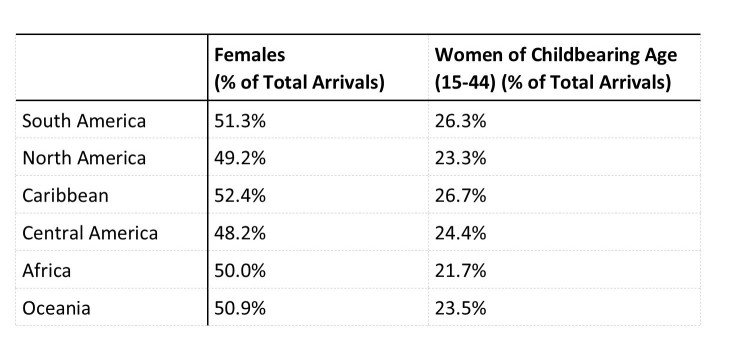
**Table 6.** Customs and Border Protection (CBP) Air Arrivals by Region to United States, Percentage of Female and Female Childbearing Age (15-44)

Applying these percentages, an estimated 17,367,810 (50.6%) of the air arrivals were women. An estimated 8,635,626 (25.2%) were women of childbearing age, and 388,603 (1.1%) were estimated to be pregnant women (Table 1).

CBP demographic data indicated that approximately 50% of all the air passenger journeys from areas with local Zika virus transmission were completed by U.S. citizens. These data underestimate arrivals by people residing in the United States who, in addition to U.S. citizens, include permanent U.S. residents, long term visitors, students, and holders of other visa types. Beyond these estimates, we are not able to determine how many travelers remain in the United States and seek medical care.

**Land Travel**

From January through December 2014, a total of 173,260,603 passenger journeys were made to the United States from Mexico by land. Among these land-crossing arrivals, 129,243,838 (75%) traveled by personal vehicle, 41,223,292 (24%) were made by pedestrians, 2,782,895 (2%) traveled by bus, and 10,500 (<1%) traveled by train (Table 1). The top four land border crossings from Mexico to the United States, regardless of mode of transportation, were San Ysidro, California, Otay Mesa, California, El Paso, Texas, and Laredo, Texas. (Fig. 2).

**Mexico-U.S. Land Border Crossing Travel Volume, 12 Month Estimate:**
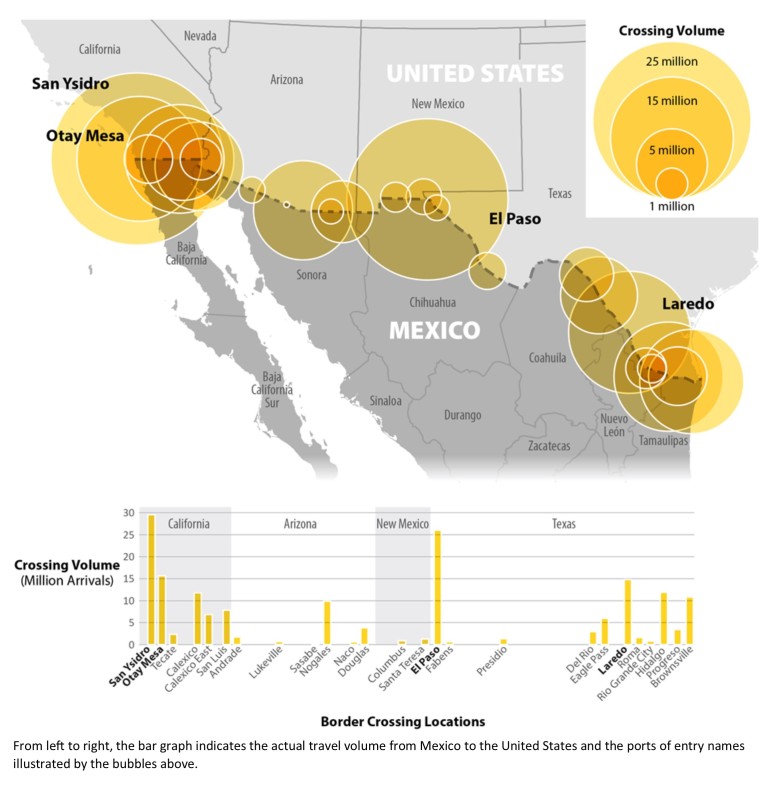

CBP land arrival demographic percentages were applied and among the 173,260,603 land-crossing arrivals entering the United States from Mexico, an estimated 82,314,319 (47.5%) were women, an estimated 40,728,656 (23.5%) were women of childbearing age, and 1,832,789 (1%) were estimated to be pregnant (Table 1).

Of the total arrivals by land, an estimated 55% were Mexican citizens, 41% were U.S. citizens, and 4% were of other or unknown citizenship. Similar to above, because of the limited data, we are not able to determine how many travelers remain in the United States and seek medical care.

**Sea Travel**

From October 2014 through September 2015, a total of 8,767,867 passenger journeys originated at a U.S. seaport and then visited a seaport in an area with local Zika virus transmission before returning to the origin U.S. seaport (Table 1). Most passenger journeys originated from and returned to ports located in Florida; specifically, Miami and Port Everglades (Table 7).

**Figure:**
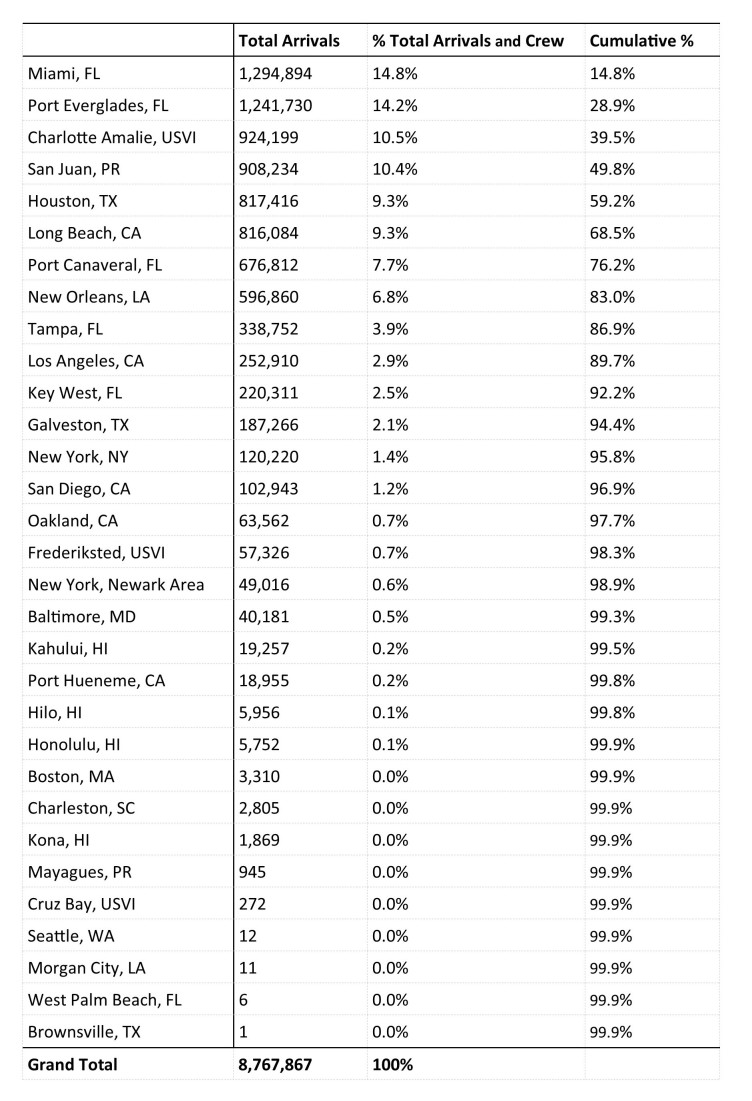
**Table 7.** U.S. Seaports of Origin with Destinations in Countries and U.S. Territories with Local Zika Virus Transmission, 12 Month Estimate

The top five Zika-impacted countries and U.S. territories visited while cruising included Mexico, Saint Maarten, Saint Thomas (U.S. Virgin Islands), Jamaica, and Haiti. Together, these seaports accounted for 78% of the total passenger journeys between U.S. seaports and areas with local Zika virus transmission (Table 8).

**Figure:**
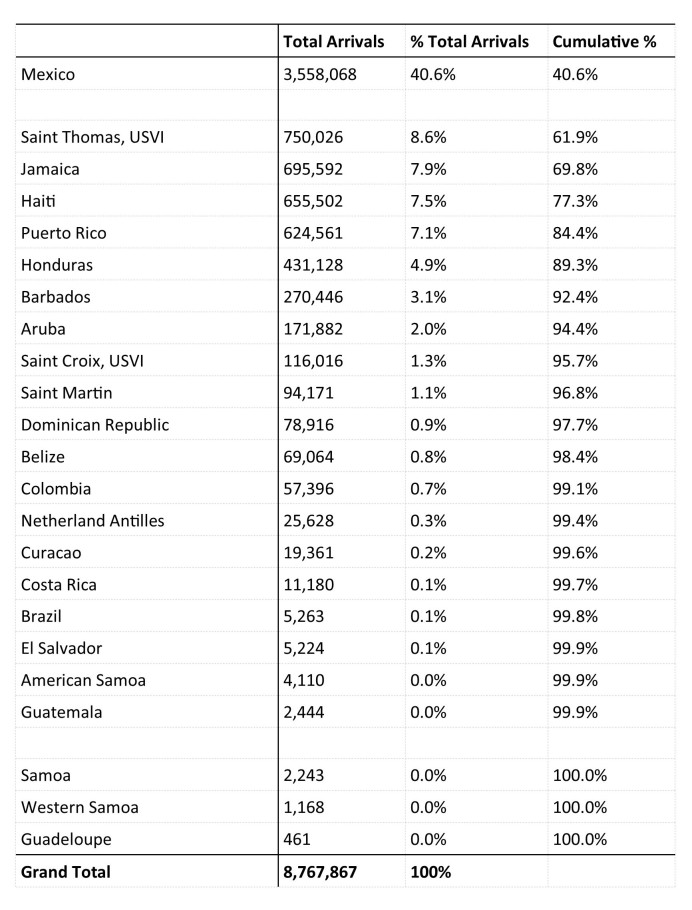
**Table 8.** Seaports Visited in Countries and U.S. Territories with Local Zika virus Transmission During Cruises from U.S. and U.S. Territories, 12 Month Estimate

Applying CBP sea arrival demographic percentages to the total 8,767,867 passenger journeys, we estimated that 4,603,130 (52.5%) were completed by women, an estimated 2,314,020 (26.4%) were completed by women of childbearing age, and 105,346 (1.2%) were estimated to have been completed by pregnant women (Table 1).

## DISCUSSION

Our analysis found that an estimated 216.3 million passenger journeys (Table 1) are made to the United States each year from areas with local Zika virus transmission. This represents roughly 60% of the total annual travel volume (358.8 million passenger journeys) into the United States from all international destinations. The destination states with the largest numbers of arrivals from areas with local Zika virus transmission include Texas (by land) and Florida (by air and sea). The origin countries with the largest number of air and land departures include Mexico, Puerto Rico, Dominican Republic, Brazil and Jamaica. Mexico, Sint Maarten, Saint Thomas (U.S. Virgin Islands), Jamaica, and Haiti account for the top five Zika-impacted countries and U.S. territories visited while cruising. Finally, we estimated that 51.7 million of the total passenger journeys are completed by women of childbearing age and 2.3 million by pregnant women.

At the global level, travel restrictions have an impact on traffic and trade. Therefore, recommending delay or cancellation of travel must reflect sensitivity and specificity, particularly when addressing a travel volume that is a large proportion of a total country’s international travel, as we presented here. This aligns with the International Health Regulations (2005) (IHR), a global treaty ratified by the World Health Assembly. The IHR’s stated goal is “to prevent, protect against, control and provide a public health response to the international spread of disease in ways that are commensurate with and restricted to public health risks, and avoid unnecessary interference with international traffic and trade”^12^. Our efforts to estimate the proportion of travelers at higher risk, particularly women of childbearing age (51.7 million) and pregnant women (2.3 million), have provided an example of narrowing the focus to decrease potential traffic and trade burden. Those estimates represent 14.4% and 0.6%, respectively, of the total annual travel volume (358.8 million passenger journeys) into the U.S. from all international destinations.

At the state and local level, public health interventions directed towards travelers can mitigate the spread of emerging infectious diseases^13^. Travel volume analyses can help identify geographic and population targets for effective health interventions. For example, prevention messaging and health education can be implemented at high volume ports of entry such as those located in Florida and Texas. Similarly, efforts to estimate high risk travelers such as women of childbearing age and those who may be pregnant, can more effectively target resources and build capacity in focused areas. Lastly, travel volume provides an input value for estimating the likelihood and distribution of potential Zika virus emergence and transmission in the United States.

This study is subject to several limitations. First, travel data are based on passenger journeys and do not indicate individual passengers. Origin and final destination city and state do not necessarily indicate the actual residence of travelers. For aviation estimates, Diio Mi FMg data as well as Department of Transportation origin and destination data, do not include passenger demographics. Estimates, therefore, were based on aggregate CBP statistics by region limiting the specificity of gender and age groups. This also limits the specificity of our estimates of pregnant women. We were not able to obtain all citizenship or residence information on arrivals, limiting our ability to estimate how many travelers remain in the United States and seek medical care. Additionally, because information about the age and gender of sea travelers were not available, we applied the air estimates for age and gender to determine the sea estimates of women, women of childbearing age, and pregnant women knowing that the populations likely differ. We do not have enough information to indicate in which direction this would bias our findings.

We foresee a continued increase of travel associated Zika virus cases in the United States. As such, travel volume analyses, such as this one, can help identify the populations and locations most likely to be affected by Zika virus; allowing for an anticipatory domestic response.

## Data Availability Statement

Data that was cited as Diio, LLC is third party owned and noted in the manuscript. Sharing in any other format beyond this manuscript is not authorized beyond provisions in our contract. There is no exclusive agreement to access the data. For researchers, the opportunity to access the data would be through the procurement of Diio Mi FMg. Please contact Diio's Senior Vice President, Jordan Kayloe, at jordan.kayloe@diio.net. Where cited as CBP, data is United States Government (USG) controlled information and due to legal restrictions may not be shared beyond provision of this manuscript without explicit written permission. Written requests for information may be submitted to DHS-SPS-RFI@hq.dhs.gov.

## Competing Interests

The authors have declared that no competing interests exist.
